# Risk Factors and Brain Metabolic Mechanism of Sleep Disorders in Autoimmune Encephalitis

**DOI:** 10.3389/fimmu.2021.738097

**Published:** 2021-11-24

**Authors:** Xiao Liu, Tingting Yu, Xiaobin Zhao, Ping Yu, Ruijuan Lv, Chunxue Wang, Lin Ai, Qun Wang

**Affiliations:** ^1^ Department of Neurology, Beijing Tiantan Hospital, Capital Medical University, Beijing, China; ^2^ China National Clinical Research Center for Neurological Diseases, Beijing, China; ^3^ Department of Nuclear Medicine, Beijing Tiantan Hospital, Capital Medical University, Beijing, China; ^4^ Department of Neuropsychiatry and Behavioral Neurology and Clinical Psychology, Beijing Tiantan Hospital, Capital Medical University, Beijing, China; ^5^ Beijing Institute of Brain Disorders, Collaborative Innovation Center for Brain Disorders, Capital Medical University, Beijing, China

**Keywords:** autoimmune encephalitis, sleep disorders, risk factors, positron emission tomography, brain metabolism, pathological mechanism

## Abstract

**Background:**

Sleep disorders (SDs) in autoimmune encephalitis (AE) have received little attention and are poorly understood. We investigated the clinical characteristics, risk factors, and cerebral metabolic mechanism of SD in AE.

**Methods:**

Clinical, laboratory, and imaging data were retrospectively reviewed in 121 consecutively patients with definite AE. The risk factors for SD in AE were estimated by logistic regression analysis. Group comparisons based on ^18^F-fluorodeoxy-glucose positron emission tomography (^18^F-FDG-PET) data were made between patients with and without SD, to further analyze potential brain metabolic mechanism of SD in AE.

**Results:**

A total of 52.9% patients (64/121) with SD were identified. The multivariate logistic model analysis showed that smoking [odds ratio (OR), 6.774 (95% CI, 1.238–37.082); *p* = 0.027], increased Hamilton Depression scale (HAMD) score [OR, 1.074 (95% CI, 1.002–1.152); *p* = 0.045], hyperhomocysteinemia [OR, 2.815 (95% CI, 1.057–7.496); *p* = 0.038], elevated neuron-specific enolase (NSE) level [OR, 1.069 (95% CI, 1.007–1.135); *p* = 0.03] were independently correlated with higher risk of SD in AE patients. Contrastingly, high MoCA score [OR, 0.821 (95% CI, 0.752–0.896); *p* < 0.001] was associated with lower risk of SD in AE subjects. Compared to controls, AE patients had less total sleep time, less sleep efficiency, longer sleep latency, more wake, higher percent of stage N1, lower percent of stage N3 and rapid eye movement, and more arousal index in non-rapid eye movement sleep (*p* < 0.05 for all). Voxel-based group comparison analysis showed that, compared to patients without SD, patients with SD had increased metabolism in the basal ganglia, cerebellum, brainstem, median temporal lobe, thalamus, and hypothalamus [*p* < 0.05, false discovery rate (FDR) corrected]; decreased metabolism in superior frontal gyrus, medial frontal gyrus, and posterior cingulate cortex (*p* < 0.001, uncorrected). These results were confirmed by region of interest-based analysis between PET and sleep quality.

**Conclusion:**

Smoking, increased HAMD score, hyperhomocysteinemia, and elevated NSE level were correlated with higher risk of SD. High MoCA score was associated with lower risk of SD in AE subjects. Moreover, a widespread metabolic network dysfunction may be involved in the pathological mechanism of SD in AE.

## Introduction

In most patients of autoimmune encephalitis (AE), the common clinical features include seizures, cognitive deficits, psychosis, and abnormal behaviors (1). In addition to these classical characteristics, there are other significant symptoms that have not been described in detail, such as sleep disorders (SDs). Although some studies recently demonstrated that SD were relatively frequent, and could lead to a poor prognosis for AE patients, SDs in patients with AE have still not received more attention (2–4). Recent studies have shown that the sleep features of AE mainly include insomnia, hypersomnolence, rapid eye movement (REM) sleep behavior disorder (RBD), and sleep apnea (5–9). However, only a limited number of cases indicate SD characteristics of AE. Moreover, the risk factors and potential pathological mechanisms of SD in AE remain unknown. Prior studies have investigated that SD tend to occur in neurological diseases, such as stroke, neurogenerative disorders (10, 11). Nevertheless, little information about risk factors of SD is available for AE patients; therefore, we hypothesized that common risk factors would be associated with SD of AE.

Furthermore, regarding pathological mechanism of SD, neuroimaging methods can be used to clarify whether SDs are related to corresponding alterations in brain structure or functional activity. ^18^F-fluoro-2-deoxy-d-glucose positron emission tomography (^18^F-FDG-PET) is a functional imaging modality for *in vivo* evaluation of the pathophysiology of the brain *via* application of ^18^F-FDG (12). Previous studies have shown that ^18^F-FDG-PET has a high sensitivity in the diagnosis of AE patients (13, 14). Meanwhile, patients with sleep disturbances showed abnormal metabolism in the brain regions that modulate sleep (15, 16). However, to the best of our knowledge, there is no systematically relevant study to evaluate the brain metabolic mechanisms of SD in patients with AE. With the aim of recognizing SD in AE, we reported 121 patients with a definite diagnosis of autoimmune encephalitis, focusing on the risk factors and brain metabolic mechanism of SD in AE.

## Materials and Methods

### Patients

This study was approved by the Ethics Committee of Beijing Tiantan Hospital that was affiliated to the Capital Medical University of the People’s Republic of China. The study was conducted in accordance with the Declaration of Helsinki, and all patients provided informed consent for the use of their medical records.

Patients were consecutively recruited and retrospectively analyzed from October 2014 to June 2021 at the Department of Neurology, Beijing Tiantan Hospital, Capital Medical University. The inclusion criteria in this study were as follows. First, patients had definite autoimmune encephalitis; the clinical diagnosis of definite AE should meet the following criteria: (a) subacute onset (rapid progression of <3 months) of working memory deficits, seizures, or psychiatric symptoms suggesting involvement of the limbic system; (b) bilateral brain abnormalities on MRI highly restricted to the medial temporal lobes; (c) cerebrospinal fluid (CSF) pleocytosis (white blood cell count of more than five cells per mm^3^); and (d) electroencephalogram (EEG) with epileptic or slow-wave activity involving the temporal lobes. The diagnosis can be made when all four criteria have been met, and final diagnosis was confirmed by the detection of serum or CSF positive for specific neuronal autoantibodies, including classical paraneoplastic antibodies (Hu, Yo, Ri, Ma2, CV2, and Amphiphysin), N-methyl-D-aspartate receptor (NMDAR), leucin-rich glioma-inactivated-1 (LGI1), contactin-associated protein-2 (CASPR2), γ-aminobutyric acid type B receptor (GABA_B_R), α-amino-3-hydroxy-5-methyl-4-isoxazolepropionic acid receptor (AMPAR), and glutamic acid decarboxylase 65 (GAD65). Serum and CSF samples were tested using both cell-based assays (Euroimmun, Lübeck, Germany) and immunohistochemical analyses in the Neuroimmunology Laboratory of the Peking Union Medical College Hospital (Beijing, China). Second, patients should be first admitted in our department from symptom onset. The exclusion criteria included (1) patients did not fulfill sleep assessment or received medication treatments before sleep assessment; (2) patients missed ^18^F-FDG-PET data; (3) patients had concurrent autoantibodies (two or more); and (4) patients had history of sleep disorder before disease onset.

### Data Collection

Clinical information on demographics, medical history, smoking, drinking, days from onset to diagnosis, comorbid symptoms, neuropsychological and psychiatric test, accompanied tumor, in-hospital laboratory test, electroencephalogram (EEG) and imaging examination, and treatments, were obtained from electronic medical records at first admission. Body mass index (BMI) was calculated as weight (kg)/height (m^2^). Neuropsychological test contained Montreal Cognitive Assessment (MoCA) (17). Depression and anxiety were evaluated using the Hamilton Depression scale (HAMD) and the Hamilton Anxiety scale (HAMA). Laboratory test included specific antibodies; white blood cell counts, proteins, and oligoclonal bands in CSF; homocysteine (hyperhomocysteinemia was defined as homocysteine concentration > 15 μmol/L); neutrophil-to-lymphocyte ratio (NLR) was calculated by dividing the absolute neutrophil count by the absolute lymphocyte count; neuron-specific enolase (NSE); and serum sodium. Imaging examination included magnetic resonance imaging (MRI) and ^18^F-FDG-PET.

### Sleep Assessment

Sleep assessment was performed through face-to-face or telephone interview from all patients or their caregivers. First, sleep complaints should be obtained, including insomnia, parasomnia, sleep breathing disorders, sleep-related behaviors or movements, and hypersomnolence. In addition, sleep quality was assessed with the standardized Pittsburgh Sleep Quality Index (PSQI) questionnaire, which evaluates multiple dimensions of sleep over a 1-month period (18). It is a self-reported questionnaire that has seven components, including subjective sleep quality, sleep latency, sleep duration, habitual sleep efficiency, sleep disturbances, use of sleeping medications, and daytime dysfunction. Each of the seven components is scored according to levels 0–3, and each score is eventually accumulated to yield a total PSQI score ranging from 0 to 21, where the higher the score, the worse the sleep quality. In this study, PSQI scores >5 indicated that patients had SD. Then, included AE patients were further divided into two subgroups (AE with SD and AE without SD) according to their PSQI results.

Only 28 patients underwent nocturnal video-polysomnography (PSG) examinations. PSG was conducted using an integrated digital recording system (Greal, Compumedics, Melbourne, Australia) that contained data acquisition, storage, and sleep analysis at the Sleep Center of Beijing Tiantan Hospital, Capital Medical University, China. Every PSG recording contained multiple channels, mainly including electrooculogram (EOG), electromyogram (EMG), electrocardiogram (ECG), respiratory signals, and EEG. EEG channels covered three brain regions (frontal, central, and occipital). Equipment placement, sleep staging, and event scoring were completed by registered polysomnographic technician. Subsequently, the following PSG parameters were collected: total time in bed; total sleep time (TST); sleep latency; sleep efficiency; wakefulness after sleep onset; percentage of stage N1, N2, N3, and rapid eye movement (REM); arousal index (AI) in whole sleep, non-rapid eye movement (NREM) sleep, and REM sleep; apnea hypopnea index (AHI); limb movements index (LMI); and periodic limb movements index (PLMI). In addition, 18 age- and gender-matched controls were recorded using the same procedures. The above variables were measured by two experienced sleep specialists blinded to clinical information of the conditions of either patients or controls, and in case of obvious discordance in their initial evaluations, an informed consensus statement was reached. The Kappa coefficient of two specialists was 0.82. REM sleep behavior disorder (RBD) was defined as a parasomnia with dream-enactment behaviors occurring during REM sleep and associated with the lack of the physiological REM sleep muscle atonia. Obstructive sleep apnea (OSA) was defined as an AHI of five or more events per hour.

### ^18^F-FDG-PET Procedure and Quantitative Analysis

All raw data of PET images were collected from PET/CT workstation; a detailed ^18^F-FDG-PET procedure has been published elsewhere using a PET/CT scanner (Elite Discovery, GE HealthCare, USA) (19). Before examination, patients did not receive neuroleptic drugs, fasted for at least 6 h, and controlled fasting blood glucose levels <8 mmol/L. Then, patients were injected with ^18^F-FDG at a dose of 3.7–5.0 MBq/kg in a quietly dedicated room. After a 45–60-min uptake period, brain PET scan was performed in the 3D-time-of-flight mode for 10 min, and whole-body PET scan was performed for approximately 20–30 min. The brain imaging data were reconstructed using ordered subset expectation maximization methods, with four iterations and eight subsets, and smoothing with a 5-mm full-width at half-maximum filter. Statistical parametric mapping (SPM) 12 (Wellcome Centre for Human Neuroimaging, London, UK) implanted in a MATLAB 2018a environment (MathWorks, Inc., USA) was used for imaging reprocessing analysis. The preprocessing steps were as follows: first, the PET images were spatially normalized into a common Montreal Neurological Institute (MNI) atlas anatomical space following a 12-parameter affine transformation and nonlinear transformations, yielding images composed of 2 mm × 2 mm × 2 mm voxels. Then, default SPM smoothing was applied using a 14-mm Gaussian kernel to increase the signal-to-noise ratio.

For the analysis on brain metabolic mechanism of sleep disorders in AE, we carried out group-level comparison between AE with SD and without SD by two-sample t-test model of SPM12 with age and gender as the nuisance variables. Significant results were viewed at the height threshold (*p* < 0.001) and corrected for multiple comparisons (FDR, corrected, *p* < 0.05). If no cluster of significant difference was found, the more liberal threshold at *p* < 0.001 uncorrected was considered to perform further exploratory analyses (20). Finally, we used the xjView SPM extension (Cui & Li, Human Neuroimaging Lab, Baylor College of Medicine) to visualize the corresponding anatomic locations of each peak MNI of these significant different clusters.

To further explore homogeneity of regional brain glucose metabolic changes between groups in particular spatial pattern, we performed a volumetric region of interest (ROI) analysis in the significant clusters based on aforementioned SPM results. First, the following 10 ROIs were identified: medial temporal lobe (MTL, mainly including amygdala and hippocampus), posterior cingulate cortex (PCC), basal ganglia (BG, including globus pallidus, putamen, and caudate), brainstem (including midbrain, pons, and medulla), superior frontal gyrus (SFG) and medial frontal gyrus (MFG), thalamus, hypothalamus, cerebellum anterior lobe (CAL), and cerebellum posterior lobe (CPL). These ROIs were defined based on the group-level results obtained from SPM analysis. The brainstem, hypothalamus, and CAL and CPL regions of interest were generated using the WFU-Pickatlas toolbox for SPM12 based on Talairach Daemon lobars, and the remaining ROIs were derived and summarized from the Anatomical Automatic Labeling (AAL) atlas. Subsequently, the ratio of the standardized uptake value (SUVR) representing ^18^F-FDG uptake calculates in those selected ROIs was obtained from each individual. Briefly, an SUVR was derived for ^18^F-FDG-PET from the voxel size weighted median uptake in the regions of interest normalized to the whole brain.

### Statistical Analysis

Continuous variables with a normal distribution were presented as the mean ± standard deviation, and non-normal variables were reported as the median [interquartile range (IQR)]. The normality of the data was performed by the Shapiro–Wilk test. Categorical variables were showed as frequency with corresponding percentage. We compared groups using t-tests for continuous variables that were normally distributed, Mann–Whitney U tests for non-parametric data, and χ² tests or Fisher’s exact tests for categorical variables.

Clinical variables were comprehensively collected for possible inclusion into the risk model. We used binary logistic regression to assess independent risk factors associated with AE with SD; all variables with *p* < 0.2 in the univariate analysis were included in multivariable logistic regression model. Subsequently, the likelihood ratio test was used in a backwards elimination process (*p* < 0.05 to retain, *p* > 0.1 to remove) to select the final set of independent risk factors for retention into the model. Odds ratio (OR) with 95% CI was presented for logistic regression model. The correlation between the SUVR values of selected ROIs and PSG parameters was conducted using Spearman test. A two-sided *p* < 0.05 was considered to indicate statistical significance. SPSS 22.0 software package (IBM Corp., Armonk, New York, USA) and Prism 8 (GraphPad Software, CA, USA) were used for statistical analyses.

## Results

### Patient Characteristics

**Figure 1:**
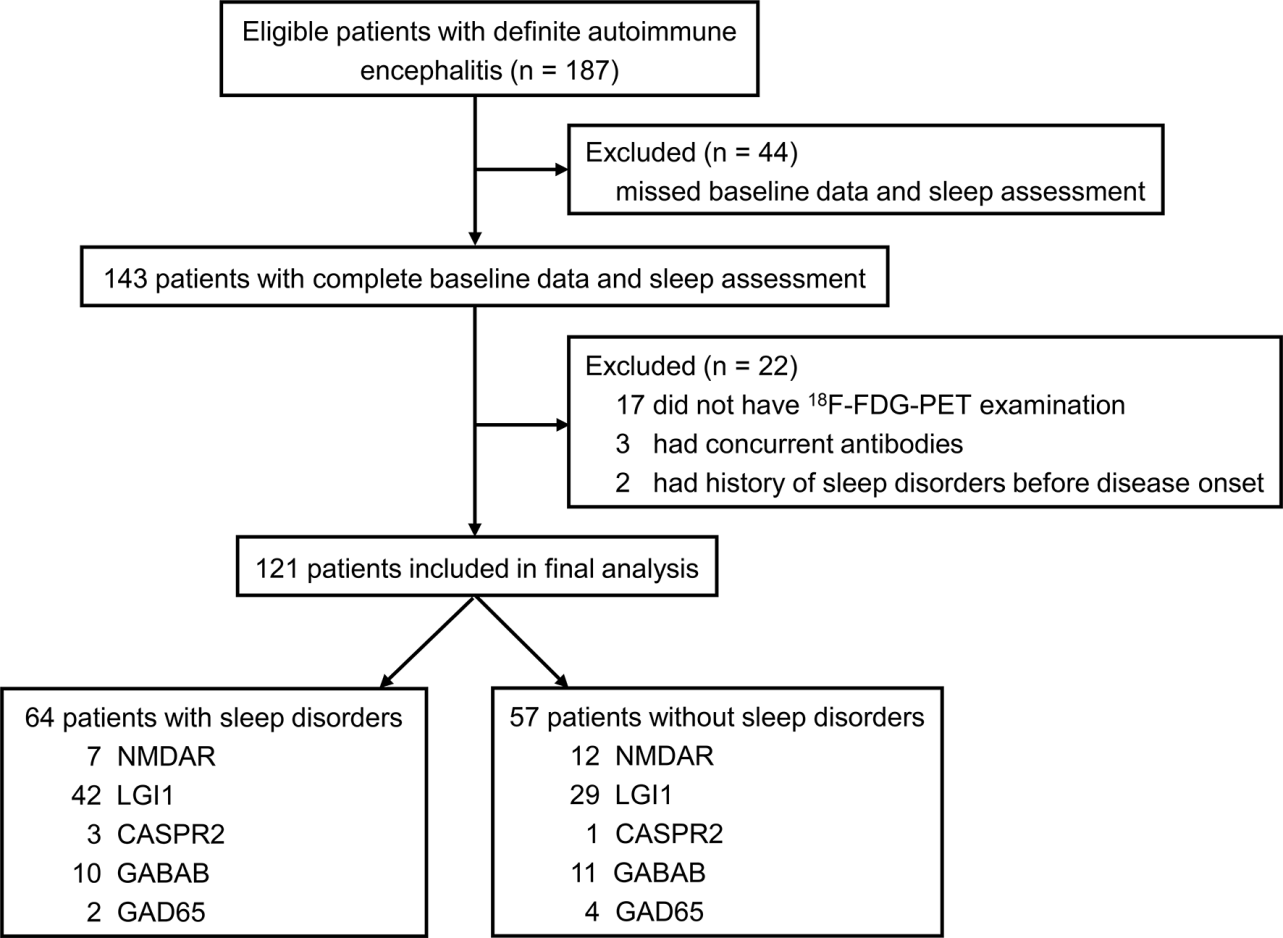
Flowchart of patient selection. ^18^F-FDG-PET, ^18^F-fluorodeoxy-glucose positron emission tomography; NMDAR, N-methyl-D-aspartate receptor; LGI1, leucin-rich glioma inactivated-1; CASPR2, contactin-associated protein-2; GABAB, γ-aminobutyric acid type B; AMPAR, α-amino-3-hydroxy-5-methyl-4-isoxazolepropionic acid receptor; GAD65, glutamic acid decarboxylase 65.

**Figure 2:**
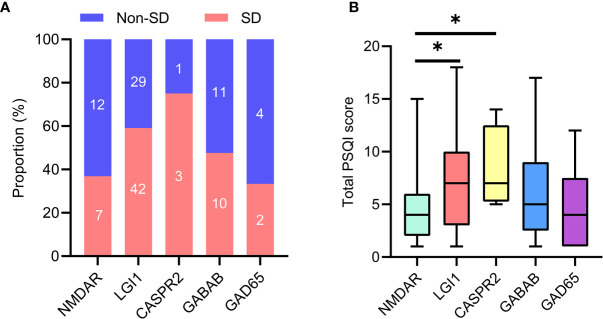
Sleep disorders in specific antibody subtypes of AE. **(A)** Number of patients with sleep disorders in subtypes of AE. **(B)** Comparison of total PSQI score was made among subtypes of AE. AE, autoimmune encephalitis; PSQI, Pittsburgh sleep quality index. *P <0.05.

The flowchart of patient inclusion is shown in **Figure 1**. A total of 187 patients with definite and antibody-confirmed AE were eligible to participate in this study, of whom 121 patients were ultimately identified in the current study after exclusion, including 19 patients with NMDAR antibodies, 71 patients with LGI1 antibodies, 4 patients with CASPR2 antibodies, 21 patients with GABA_B_R antibodies, and 6 patients with GAD65 antibodies. Among these cases, 52.9% of AE patients (n = 64) had SD (36.8% NMDAR, 59.2% LGI1, 75% CASPR2, 47.6% GABA_B_R, and 33.3% GAD65; **Figure 2A**); the median PSQI score in the group of SD was 9 (IQR, 7–14), which was obviously higher than those without SD [median, 9 (IQR, 7–14) vs. 3 (IQR, 2–3), *p* < 0.001). In addition, patients with CASPR2 and LGI1 had higher PSQI score than those with NMDAR antibodies (*p* < 0.05, **Figure 2B**).

There is no significant difference regarding the median time from diagnosis to the sleep assessment [9 (IQR, 6–20) days vs. 9 (IQR, 6–17) days, *p* = 0.892) between patients with SD and without SD. The baseline characteristics of patients based on sleep quality are summarized in **Table 1**. Patients with SD were older than those without SD (median age, 55.4 vs. 49.0 years, *p* =0.001). Hypertension, smoking, cognitive deficits, psychosis, and abnormal behaviors showed statistically differences between patients with SD and without SD (*p* < 0.05 for all). Compared with patients without SD, patients with SD had higher levels of MoCA score (*p* =0.001) and NSE (*p* < 0.001) and had lower levels of serum sodium (*p* =0.001). Eleven of these patients (9%) were associated with tumors: 8 lung cancers (2 with LGI1 antibodies, 7 with GABAB antibodies), 1 colorectal adenoma (1 with LGI1 antibodies), 1 ovarian teratoma (1 with NMDAR antibodies), and 1 thymoma (1 with LGI1 antibodies). Tumor removal was performed in three patients, and chemotherapy was performed in eight patients. In a univariate analysis, seven patients (10.9%) presented tumors in the group of sleep disorders, and four patients (7%) with tumor were shown in the group of patients without sleep disorders. There was no statistical significance between the two groups (*p* = 0.454). All patients received first-line immunotherapy [20 IV methylprednisolone (IVMP) alone, 21 IV immunoglobulins (IVIg) alone, and 80 IVMP + IVIg]. Twelve patients additionally received second-line immunotherapy (four rituximab and eight mycophenolate mofetil). There was no significant difference between patients with SD and without SD regarding relevant treatments (*p* = 0.363). More details of corresponding descriptions are reported in **Table 1**.

**Table 1:** Baseline characteristics of AE patients with and without SD.

Characteristics	All patients (n = 121)	Patients with SD (n = 64)	Patients withoutSD (n = 57)	*p-*value
Age, median (IQR), years	54 (37–64)	55.4 (44.3–67.0)	49.0 (31.5–60.0)	0.001
Male, n (%)	77 (63.6)	45 (70.3)	32 (56.1)	0.106
Body mass index, mean (standard deviation)	24.5 (3.9)	24.4 (3.6)	24.5 (4.2)	0.877
Medical history, n (%)				
Hypertension	31 (25.6)	22 (34.4)	9 (15.8)	0.019
Diabetes	11 (9.1)	6 (9.4)	5 (8.8)	0.908
Smoking	33 (27.3)	23 (35.9)	10 (17.5)	0.023
Drinking	28 (23.1)	18 (28.1)	10 (17.5)	0.168
Comorbid symptoms, n (%)				
Cognitive deficits	92 (76.0)	54 (84.4)	38 (66.7)	0.023
Seizures	114 (94.2)	61 (95.3)	53 (93.0)	0.706
Psychosis and abnormal behaviors	71 (58.7)	46 (71.9)	25 (43.9)	0.002
Movement disorders	11 (9.1)	5 (7.8)	6 (10.5)	0.604
Duration of symptoms, median (IQR), days				
Cognitive deficits	235 (133–345)	233 (147–344)	255 (127–348)	0.978
Seizures	72 (37–189)	72 (46–182)	71 (28–191)	0.672
Psychosis and abnormal behaviors	85 (62–123)	88 (61–125)	87 (66–120)	0.918
Movement disorders	115 (63–284)	70 (46–343)	159 (55–294)	0.522
MoCA score, median (IQR)	20 (15–24)	17 (14–20)	24 (18–26)	<0.001
HAMA score, median (IQR)	10 (4.5–14.5)	10.5 (5.0–16.5)	10 (4.0–13.5)	0.466
HAMD score, median (IQR)	12 (6–17)	14.0 (5.3–18.0)	11 (6–16)	0.093
Tumor, n (%)	11 (9.1)	7 (10.9)	4 (7.0)	0.454
Specific antibody test, n (%)				0.288
NMDAR	19 (15.7)	7 (10.9)	12 (21.1)	
LGI1	71 (58.7)	42 (65.6)	29 (50.9)	
CASPR2	4 (3.3)	3 (4.7)	1 (1.8)	
GABAB	21 (17.4)	10 (15.6)	11 (19.3)	
GAD65	6 (5.0)	2 (3.1)	4 (7.0)	
CSF at first admission, n (%)				
CSF pleocytosis^a^	49 (40.5)	28 (43.8)	21 (36.8)	0.440
Elevated protein concentration^b^	32 (26.4)	20 (31.3)	12 (21.1)	0.204
Positive oligoclonal bands	54 (44.6)	28 (43.8)	26 (45.6)	0.837
Hyperhomocysteinemia, n (%), μmol/L^c^	41 (33.9)	26 (40.6)	15 (26.3)	0.097
NLR, median (IQR)^d^	2.6 (2.1–3.7)	2.8 (2.1–4.1)	2.4 (1.9–3.2)	0.062
NSE, median (IQR), μg/L	19.6(15.0–25.1)	21.4 (16.7–26.9)	18.5 (13.9–21.9)	0.019
Serum sodium, median (IQR), mmol/L	139 (130–141)	134 (127–140)	140 (136.5–141.5)	0.001
EEG, n (%)				0.922
Slow waves or epileptiform discharges in temporal region	78 (64.5)	41 (64.1)	37 (64.9)	
Others	43 (35.5)	23 (35.9)	20 (35.1)	
Initial MRI results, n (%)				0.130
Normal	72 (59.5)	34 (53.1)	38 (66.7)	
Medial temporal lesions	49 (40.5)	30 (46.9)	19 (33.3)	
Days from onset to diagnosis	54 (25–140)	50 (25.5–104.8)	59 (22.5–217.5)	0.584
Days from diagnosis to PET scans	6 (2–17)	6 (2–18)	5 (2–16)	0.698
Days from diagnosis to sleep assessment	9 (6–18)	9 (6–20)	9 (6–17)	0.892
Treatment, n (%)				
First-line immunotherapy				0.363
IVMP only	20 (16.5)	8 (12.5)	12 (21.1)	
IVIg only	21 (17.4)	13 (20.3)	8 (14)	
IVMP + IVIg	80 (66.1)	43 (67.2)	37 (64.9)	
Second-line Immunotherapy				
Rituximab	4 (3.3)	3 (4.7)	1 (1.8)	0.621
Mycophenolate Mofetil	8 (6.6)	5 (7.8)	3 (5.3)	0.721
Tumor removal	3 (2.5)	2 (3.1)	1 (1.8)	0.544
Tumor chemotherapy	8 (6.6)	3 (4.7)	5 (8.8)	0.473

AE, autoimmune encephalitis; SD, sleep disorders; IQR, interquartile range; MoCA, Montreal cognitive assessment; HAMA, Hamilton anxiety scale; HAMD, Hamilton depression scale; NMDAR, N-methyl-D-aspartate receptor; LGI1, leucin-rich glioma inactivated-1; CASPR2, contactin-associated protein-2; GABAB, γ-aminobutyric acid type B; AMPAR, α-amino-3-hydroxy-5-methyl-4-isoxazolepropionic acid receptor; GAD65, glutamic acid decarboxylase 65; CSF, cerebrospinal fluid; NLR, neutrophil-to-lymphocyte ratio; NSE, neuron-specific enolase; EEG, electroencephalogram; MRI, magnetic resonance imaging; PET, positron emission tomography; IVMP, IV methylprednisolone; IVIG,IV immunoglobulin.

a CSF pleocytosis indicates white blood cell count of more than five cells per mm³.

b Elevated protein concentration is considered when protein levels of >45 mg/dl.

c Hyperhomocysteinemia is defined as homocysteine concentration >15 μmol/L.

d NLR is calculated by dividing the absolute neutrophil count by the absolute lymphocyte count.

### PSG Findings

A subgroup of 28 patients completed PSG recordings. These 28 patients were a representative sample of the whole cohort of 121 patients because there were no significant differences across clinical variables between patients with PSG and the whole cohort (**Supplementary Table S1**).

The sleep parameters of PSG are presented in **Table 2**. The median TST was 292.8 (160–489.4) min. Sleep efficiency was approximately 56.1%. Sleep onset latency was 37 (1.5–189) min. Wakefulness after sleep onset was 145.8 (26.5–264.5) min. For percent of sleep stages, the stage N1, N2, N3, and REM comprised 25.2% (4.3%–56.8%), 51.4% (15.6%–85.6%), 9.4% (0%–29.8%), and 12.5% (0.7%–32.5%) of the TST, respectively. REM sleep latency was 139 (24–287.5) min. Nine patients had dream enhancement behaviors, and the final diagnosis of RBD was confirmed on PSG in five patients (5/28, 17.9%), of whom three had LGI1 antibodies. Sixteen of 28 patients (57.1%) developed OSA with median AHI 10/hour. AI in whole sleep, NREM, and REM were 8.6 (0–33.5), 17 (0.5–33), and 14 (0.9–30), respectively. The median LMI and PLMI were 16/h (1–360/h) and 12/h (0–25/h), respectively.

**Table 2:** PSG findings in patients with AE compared with controls.

	Patients with AE (n = 28)	Controls (n = 18)	*p-*value
Age, median (IQR), years	57 (35–66)	55 (40–63)	0.736
Male, n (%)	19 (68)	13 (72)	0.756
Body mass index	24.2 (15.9–36.3)	24.4 (17.7–34)	0.719
Total time in bed, min	522.2 (424.4–677.3)	530.4 (420.7–598.6)	0.613
Total sleep time, min	292.8 (160–489.5)	432.0 (172–528.5)	0.001*
Sleep latency, min	37 (1.5–189)	10 (0.5–93.5)	0.005*
Sleep efficiency, %	56.1 (28–94)	80.5 (41–96)	0.001*
Wakefulness after sleep onset, min	145.8 (26.5–264.5)	76.8 (18.5–237.5)	0.004*
Stage N1, %	25.2 (4.3–56.8)	10.4 (4.7–43.2)	0.002*
Stage N2, %	51.4 (15.6–85.6)	52.4 (32.7–66)	0.893
Stage N3, %	9.4 (0–29.8)	14.3 (2.3–21.8)	0.013*
Stage REM, %	12.5 (0.7–32.5)	20.8 (13.4–32.4)	0.001*
REM sleep latency, min	139 (24–287.5)	76 (4–212.5)	0.098
Arousal index in whole sleep, n/hour	8.6 (0–33.5)	11.3 (4.2–43.6)	0.207
Arousal index in NREM sleep, n/hour	17 (0.5–33)	8.9 (3.3–18.6)	0.003*
Arousal index in REM sleep, n/hour	14 (0.9–30)	8.1 (2.5–49.6)	0.306
AHI, n/hour	10 (0–52.6)	4.3 (0–43.2)	0.140
LMI, n/hour	16 (1–360)	10 (0–32.4)	0.242
PLMI, n/hour	12 (0–25)	1.5 (0–28.3)	0.111

Results are presented as median and range.

PSG, polysomnography; AE, autoimmune encephalitis; IQR, interquartile range; n, number; min, minutes; REM, rapid eye movement; NREM, non-rapid eye movement; N, NREM; AHI, apnea hypopnea index (n/hour); LMI, limb movements index (n/hour); PLMI, periodic limb movements index (n/hour). *p < 0.05.

There were no significant differences between patients with SD and without SD regarding age, gender, and body mass index (**Table 2**). Compared to controls, AE patients had less TST, less sleep efficiency, longer sleep latency, more wake, higher percent of stage N1, lower percent of stage N3 and REM, and more AI in NREM sleep (*p* < 0.05 for all). Other sleep variables demonstrated no significant differences between the two groups.

### Analysis of Risk Factors for SD in AE

Results of multivariate logistic analysis of the final model are shown in **Table 3**. We selected variables with *p* < 0.2 in the univariate analysis, including age, male, hypertension, smoking, drinking, cognitive deficits, psychosis and abnormal behaviors, MoCA score, HAMD score, hyperhomocysteinemia, NLR, NSE, serum sodium, and initial MRI. After adjusting for all included clinical variables, the logistic regression analysis showed that smoking [OR, 6.774 (95% CI, 1.238–37.082); *p* = 0.027], increased HAMD score [OR, 1.074 (95% CI, 1.002–1.152); *p* = 0.045], hyperhomocysteinemia [OR, 2.815 (95% CI, 1.057–7.496); *p* = 0.038), elevated NSE level [OR, 1.069 (95% CI, 1.007–1.135); *p* = 0.03] were independently correlated with higher risk of SD in AE patients. Contrastingly, high MoCA score [OR, 0.821 (95% CI, 0.752–0.896); *p* < 0.001] was associated with lower risk of SD in AE subjects.

**Table 3:** Multivariate logistic regression of independent factors associated with SD in AE.

Variables^a^	Regression coefficient	Odds ratio (95% CI)	*p*-value
Hypertension	1.081	2.946 (0.929–9.349)	0.067
Smoking	1.913	6.774 (1.238–37.082)	0.027
Drinking	−1.760	0.172 (0.026–1.160)	0.071
MoCA	−0.198	0.821 (0.752–0.896)	< 0.001
HAMD	0.072	1.074 (1.002–1.152)	0.045
Hyperhomocysteinemia	1.035	2.815 (1.057–7.496)	0.038
NSE	0.067	1.069 (1.007–1.135)	0.030

AE, autoimmune encephalitis; SD, sleep disorders; MoCA, Montreal cognitive assessment; HAMD, Hamilton depression scale; NSE, neuron-specific enolase.

a These are the final variables that were retained following the application of multivariable logistic regression with backwards elimination process.

### Voxel-Wise Analysis of ^18^F-FDG-PET

**Figure 3:**
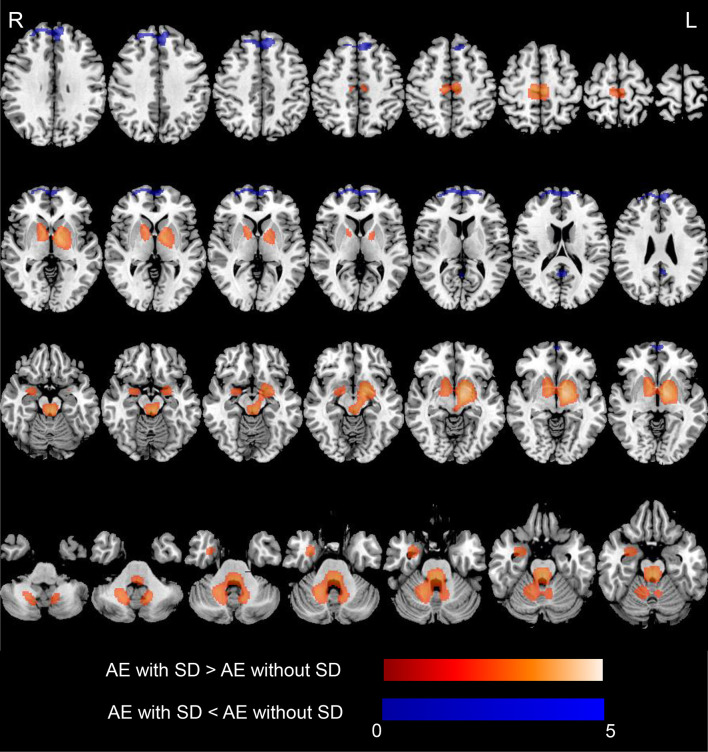
^18^F-FDG-PET results based on SPM in AE patients with SD compared with those who did not develop SD. T-maps show hypo- (blue color) and hyper-metabolism (hot color). Patients with SD demonstrated relatively increased metabolism in the MTL (amygdala, hippocampus), and basal ganglia (medial globus pallidus, putamen, caudate), extending to the cerebellum (cerebellum anterior lobe, cerebellum posterior lobe, dentate, cerebellar tonsil, culmen), brainstem (midbrain, pons), and thalamus and hypothalamus (*p* < 0.05, FDR corrected). Patients with SD showed hypometabolism in the frontal lobe (medial frontal gyrus, superior frontal gyrus), posterior cingulate cortex (*p* < 0.001, uncorrected). Axial slices are shown according to radiological convention (right is left). ^18^F-FDG-PET, ^18^F-fluorodeoxy-glucose positron emission tomography; SPM, statistical parametric mapping; AE, autoimmune encephalitis; SD, sleep disorders; R, right; L, left.

**Figure 3** shows the significant metabolic differences of group comparisons based on voxel-based analysis between patients with and without SD, no significant difference was noted between the two groups with regard to the median time from diagnosis to the initial scan [6 (IQR, 2–18) days vs. 5 (IQR, 2–16) days, *p* = 0.698]. Patients with SD demonstrated relatively increased metabolism in the MTL, BG (medial globus pallidus, putamen, and caudate), extending to the cerebellum (cerebellum anterior lobe, cerebellum posterior lobe, dentate, cerebellar tonsil, and culmen), brainstem (midbrain and pons), thalamus, and hypothalamus compared to patients without SD (*p* < 0.05, FDR corrected; **Figure 3** and **Table 4**). Furthermore, patients with SD also showed hypometabolism in the frontal lobe (MFG and SFG) and PCC (*p* < 0.001, uncorrected; **Figure 3** and **Table 4**). Cluster extent, peak MNI coordinates, corrected *p* values, maximum *Z* value, corresponding brain regions, and voxel size for each region are detailly reported in **Table 4**.

**Table 4:** ^18^F-FDG-PET metabolic differences of SPM comparison between AE with SD and without SD.

	Cluster extent	*p-*value	Maximum *Z* score	Peak MNI coordinates			Brain region	Voxel size
				x	y	Z		
AE with SD > without SD	5514	0.038^a^	4.14	−12	−4	−6	Medial globus pallidus^c^	147
							Putamen	271
							Caudate	104
							Cerebellum anterior lobe	1341
							Cerebellum posterior lobe	421
							Dentate	337
							Cerebellar tonsil	165
							Culmen	498
							Midbrain	784
							Pons	523
							Amygdala	202
							Hippocampus	71
							Para-hippocampus gyrus	291
							Uncus	123
							Thalamus	67
							Hypothalamus	12
	807	0.038^a^	3.74	8	−24	−56	Medial frontal gyrus^c^	581
							Paracentral lobule	172
AE with SD < without SD	2012	< 0.001^b^	3.88	−6	36	46	Medial frontal gyrus^c^	871
							Superior frontal gyrus	856
							Middle frontal gyrus	93
							Orbitofrontal cortex	27
	217	< 0.001^b^	3.43	−2	−52	18	Posterior cingulate cortex ^c^	191
							Precuneus	33

^18^F-FDG-PET, ^18^F-fluoro-2-deoxy-d-glucose positron emission tomography; SPM, statistical parametric mapping; AE, autoimmune encephalitis; SD, sleep disorders; MNI, Montreal Neurological Institute.

a p < 0.05 corrected for multiple comparisons with the false discovery rate.

b p < 0.001 uncorrected.

c The indicated region is the cluster’s peak region.

### Correlations Between ROI-Based FDG-PET Findings and Sleep Quality

**Figure 4:**
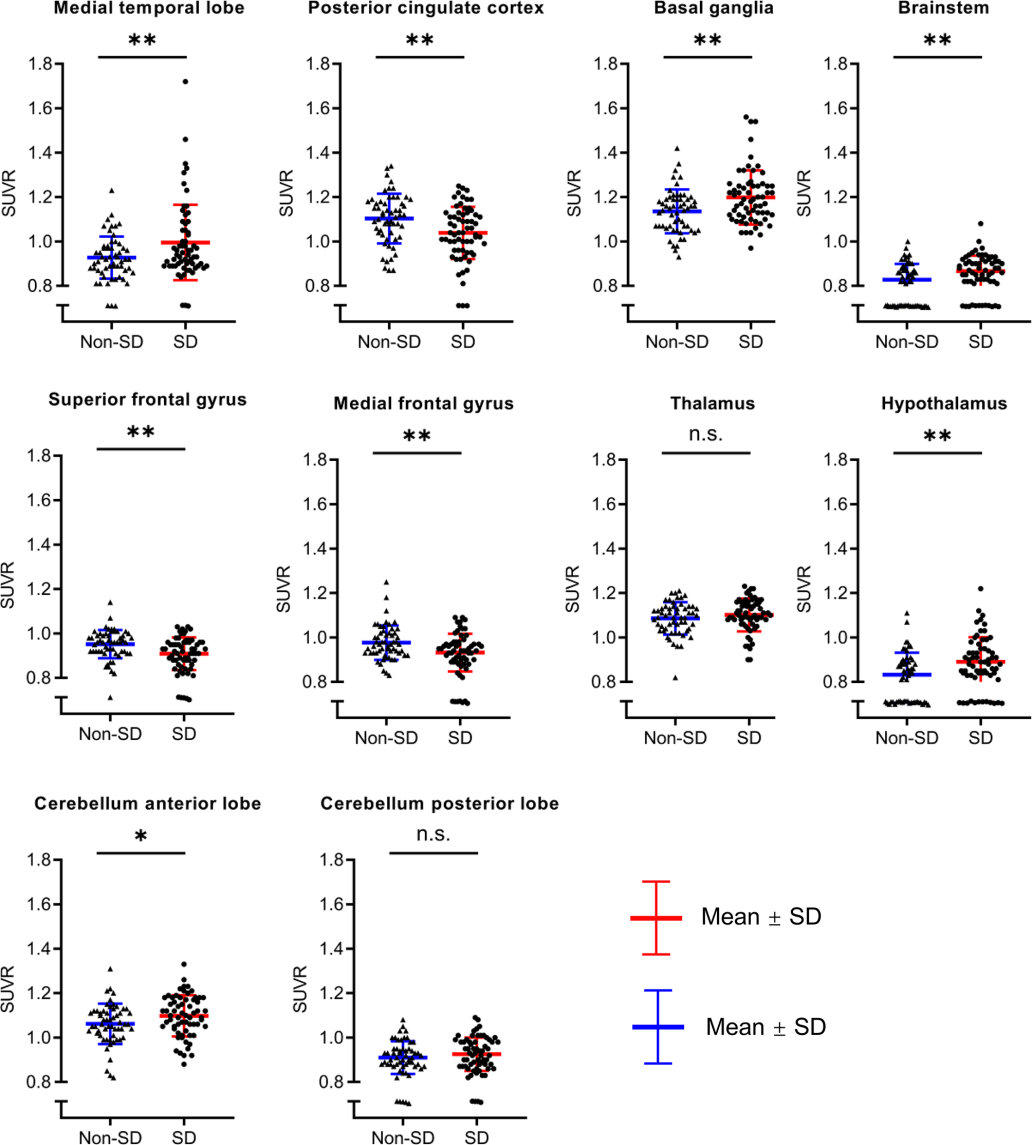
The comparison of SUVR value between AE patients with SD and without SD. Compared with patients without SD, patient with SD showed relatively higher metabolism in the medial temporal lobe, basal ganglia, brainstem, hypothalamus, cerebellum anterior lobe. Patients with SD also demonstrated more increased metabolism than those without SD in the thalamus and cerebellum posterior lobe, but there was no significant difference between two groups. In contrast, decreased metabolism in the superior frontal gyrus, medial frontal gyrus, and posterior cingulate cortex were observed in patients with SD in comparison with those without SD. SUVR, standardized uptake value ratio; AE, autoimmune encephalitis; SD, sleep disorders; ns, no significance; *p < 0.05; **p < 0.01.

To investigate and validate whether the above significant clusters containing different brain regions were truly related to sleep quality, we further conducted an ROI-based correlation analysis between patients with SD and without SD. The comparison of SUVR value between the two groups is shown in **Figure 4**. Compared with patients without SD, patients with SD showed relatively higher metabolism in the MTL (0.99 ± 0.17 vs. 0.93 ± 0.09, *p* = 0.008), BG (1.2 ± 0.12 vs. 1.14 ± 0.1, *p* = 0.002), brainstem (0.87 ± 0.07 vs. 0.83 ± 0.07, *p* = 0.003), hypothalamus (0.89 ± 0.11 vs. 0.83 ± 0.1, *p* = 0.003), CAL (1.1 ± 0.09 vs. 1.06 ± 0.09, *p* = 0.033). Patients with SD also demonstrated more increased metabolism than those without SD in thalamus (1.1 ± 0.07 vs. 1.09 ± 0.07, *p* = 0.251) and CPL (0.93 ± 0.08 vs. 0.91 ± 0.07, *p* = 0.269), but there was no significant difference between the two groups. In contrast, decreased metabolism in the SFG (0.91 ± 0.07 vs. 0.95 ± 0.06, *p* = 0.001), MFG (0.93 ± 0.08 vs. 0.98 ± 0.08, *p* = 0.003), and PCC (1.04 ± 0.12 vs. 1.1 ± 0.11, *p* = 0.002) were observed in patients with SD in comparison with those without SD.

**Figure 5:**
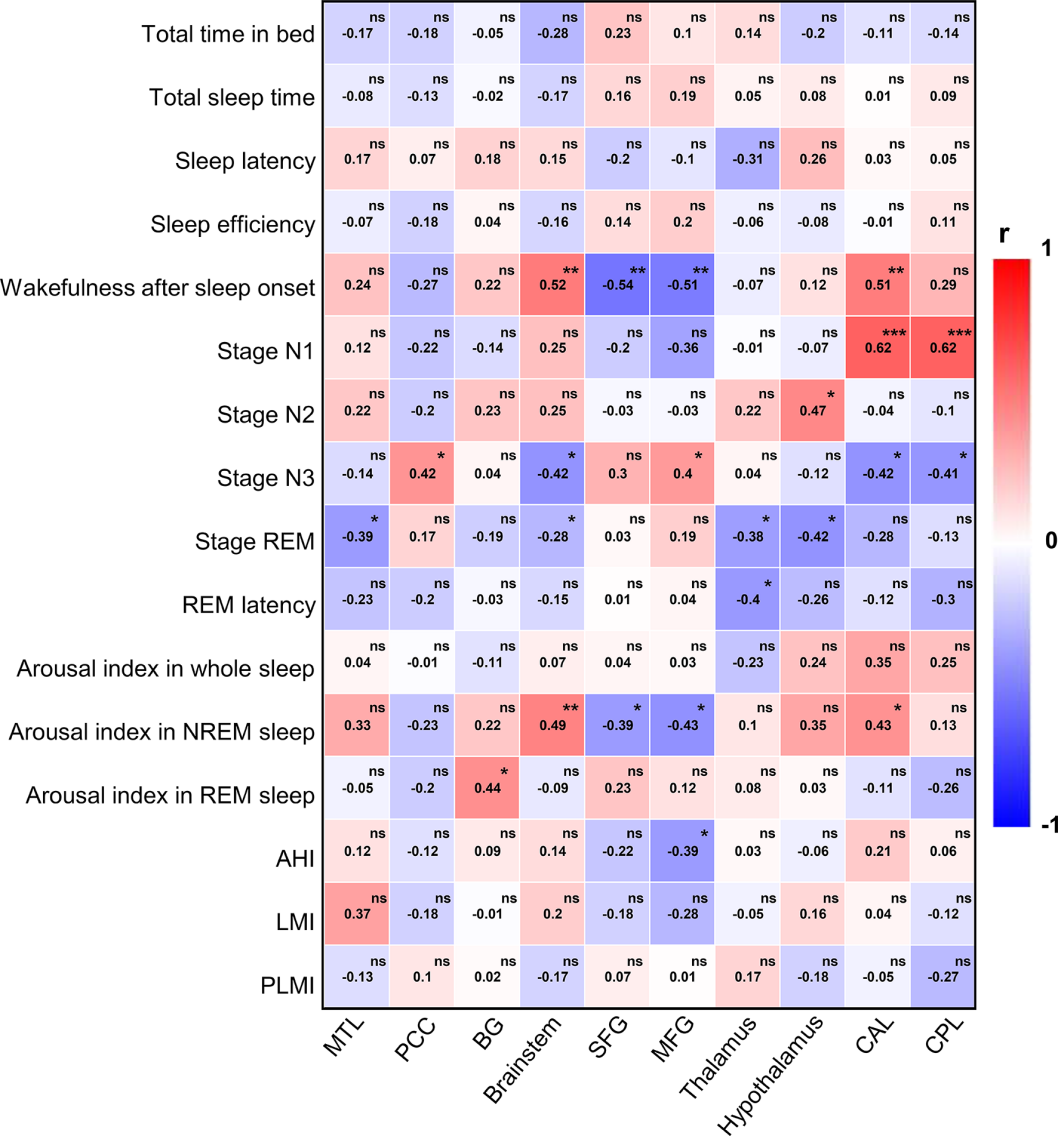
PSG-based correlation analysis between ^18^F-FDG-PET and sleep quality. PSG, polysomnography; ^18^F-FDG-PET, ^18^F-fluorodeoxy-glucose positron emission tomography; REM, rapid eye movement; NREM, non-rapid eye movement; AHI, apnea hypopnea index; LMI, limb movements index; PLMI, periodic limb movements index; MTL, medial temporal lobe; PCC, posterior cingulate cortex; BG, basal ganglia; SFG, superior frontal gyrus; MFG, medial frontal gyrus; CAL, cerebellum anterior lobe; CPL, cerebellum posterior lobe. The color bar indicates Spearman correlation coefficient and is scaled from red to blue; ns, no significant; *p < 0.05; **p < 0.01; ***p < 0.001.

In order to explore the relationship between PET and sleep quality in more detail, we further performed a more fine-grained PSG-based correlation analysis (**Figure 5**). Wakefulness after sleep onset was associated with abnormal metabolism in the brainstem (r = 0.52, *p* =0.005), SFG (r = −0.54, *p* =0.003), MFG (r = −0.51, *p* = 0.005), and CAL (r = 0.51, *p* = 0.006). Stage N1 correlated with cerebral metabolism predominantly in CAL (r = 0.62, *p <*0.001) and CPL (r = 0.62, *p <*0.001). Stage N2 was associated with metabolism in the hypothalamus (r = 0.47, *p* = 0.012). Stage N3 was related to glucose metabolism in PCC (r = 0.42, *p* =0.027), brainstem (r = −0.42, *p* = 0.026), MFG (r = 0.4, *p* = 0.033), CAL (r = −0.42, *p* = 0.028), CPL (r = −0.41, *p* = 0.029). Stage REM correlated with cerebral metabolism in MTL (r = −0.39, *p* = 0.041), brainstem (r = −0.28, *p* =0.04), thalamus (r = −0.38, *p* =0.049), and hypothalamus (r = -0.42, *p* =0.027). REM latency was associated brain metabolism in thalamus (r = -0.4, *p* =0.035). AI in NREM sleep was related to glucose metabolism in brainstem (r = 0.49, *p* =0.009), SFG (r = −0.39, *p* =0.042), MFG (r = −0.43, *p* = 0.021), CAL (r = 0.43, *p* =0.024). AI in REM sleep correlated with cerebral metabolism mainly in BG (r = 0.44, *p* = 0.021). AHI was associated with abnormal metabolism in MFG (r = −0.39, *p* = 0.041). There was no statistically significant correlation between other PSG variables and regional brain metabolism on FDG-PET.

## Discussion

In this study, we reported a cohort of 121 patients with antibody-confirmed AE and investigated the potential risk factors and metabolic mechanism for AE with SD. We found that smoking, increased HAMD score, hyperhomocysteinemia, and elevated NSE level were independently correlated with higher risk of SD. Contrastingly, high MoCA score was associated with lower risk of SD in AE subjects. In addition, compared with patients without SD, patients with SD showed increased metabolism in the basal ganglia, cerebellum, brainstem, MTL, thalamus, and hypothalamus and decreased metabolism in frontal (MFG and SFG) and PCC, suggesting that a widespread metabolic network disfunction may be involved in the pathology of SD in AE.

In recent years, many studies have shown that sleep can be evidently affected by antibody-mediated diseases. However, AE-related SDs do not attract enough attention from clinicians, and little is known about the risk factors of SD in AE. Our results demonstrated that smoking was independently associated with high risk of SD in AE. Prior study showed that high levels of tobacco exposure were associated with sleep disturbance (21), but how smoking contributes to AE-related SD remains unclear. One possible explanation was that tobacco could promote neurotransmitters disturbance (22); meanwhile, it also affected activity of B- and T-lymphocyte subsets, promoted cytokine-driven systemic inflammation, and further might act to induce autoimmune diseases (23). Therefore, when the abnormal immune reaction and neurotransmitter imbalance attack sleep-related neurons or structures may lead to sleep disorders. Understanding the interactions of these transmitter systems could be important for the development of SD in AE.

Hyperhomocysteinemia is an independent risk factor for SD in AE subjects in the current study. Although the potential mechanism through which elevated homocysteine acts to increase the high risk of AE-associated SD is unclear, the following reasons may explain this result. Homocysteine, as a multifunctional factor, has been generally reported to play a complex role in neurological disorders, such as stroke and neurodegenerative diseases (24, 25). Increased homocysteine mainly contributes to pathological vascular injury and aggravates inflammatory process (26); patients with OSA are frequently accompanied by hyperhomocysteinemia, which may be related to severe oxidative stress (27). However, the potential association between SD and increased homocysteine remains uncertain. We speculated that brain tissues suffered irreversible damage due to ischemia and hypoxia induced by elevated homocysteine and released a large number of harmful free radicals, which affected neural central structures and the dopaminergic neurons regulating sleep and further caused sleep–wake dysfunction. More attention should be paid to homocysteine and its metabolic pathways in the further study.

Furthermore, we also found that the elevated NSE was correlated with increased risk of SD in AE. NSE is an important regulatory enzyme in the process of glycolysis and is widely found in neurons and neuroendocrine cells; high levels of NSE indicate the neuron injury (28). In this study, the serum NSE level in AE with SD group was higher than that in the non-SD group, suggesting that sleep disorders were closely related to neuron damage. In addition, elevated NSE was also observed in OSA patients compared to controls (29). Thus, it is proved that central nervous system injury based on immunological derangement may be an important cause of sleep disorders.

Although it has been suggested that sleep disorders are frequent and may be influenced by impaired brain regions involved in the sleep–wake regulatory network in patients with autoimmune encephalitis, the exact neuroanatomical and pathological mechanism behind sleep disfunction caused by AE is still unknown. The current findings from the aspect of brain mentalism using ^18^F-FDG-PET indicated that, compared with patients without SD, patients with SD exhibited highly hypermetabolism in the MTL, BG, brainstem, thalamus, hypothalamus, and cerebellum and decreased metabolism in the SFG, MFG, and PCC, suggesting that AE-related SD is not confined to the limbic system but rather affects a wide range of brain regions and functional networks. Some studies showed that sleep disturbances might be related to abnormal neural network connectivity, such as the default mode network (DMN), which comprises multiple interwoven networks, and is mainly associated with memory, cognitive function, and maintenance of conscious state (30, 31). A functional MRI (fMRI) study showed that, compared with controls, patients with insomnia demonstrated increased functional connectivity (FC) between DMN and sensory-motor network and decreased FC between DMN and salience network (32). PCC and MTL, as important components of DMN, may play important roles in the regulation of sleep disorders. A prior study reported decreased ALFF values in the bilateral posterior cingulate gyrus in patients with anti-NMDAR encephalitis, which may be related to SD (33). However, functional connectivity analysis of SD in AE is lacking; future research about this should be conducted. The hypothalamus is an important sleep center involved in the regulation of sleep and wake. This may be a good explanation for SD in patients with encephalitis associated with LGI1 antibody, which is often related to hypothalamic disorders. In addition, it has been suggested that, compared with controls, AD with SD showed decreased metabolism in the hypothalamus (34). However, AE with SD exhibited hypermetabolism compared with controls, which may result from the inflammatory process in the acute stage accompanied by a rapid increase in neural antibodies and impairment of synaptic plasticity.

For PSG, AE patients had higher percent of stage N1, lower percent of stage N3, and more AI in NREM sleep than controls in the current study, which was similar to a prior study (7). Further PET-based correlation analysis showed that NREM sleep of stage N1 correlated with the cerebellum; stage N3 was related to PCC, brainstem, MFG, and cerebellum; AI in NREM sleep was related to the brainstem, SFG, MFG, and cerebellum, suggesting that cerebellum may play an important regulatory role in NREM sleep. Some studies have suggested that Purkinje cells (PCs) in the cerebellar cortex exhibited increased activity prior to the transition from sleep to wakefulness; in addition, the increased PC activity was accompanied by decreased activity in neurons of the deep cerebellar nuclei (DCN) at the NREM sleep–wakefulness transition (35). Moreover, the cerebellum might be a novel candidate for regulating sleep and/or wakefulness states *via* its interaction with arousal neurons in the ventral thalamus and hypothalamus (36). Thus, the cerebellum is at the heart of communication with arousal neurons that regulate sleep and/or arousal states.

There are several limitations in this study. (1) Due to the retrospective nature and relatively small sample size of this study, there exists a potential selection or recall bias. (2) This study measured sleep quality through subjective self-report scale; objective polysomnography can better reflect the characteristics and accurate classification of sleep disorders. However, objective results are only available in partial patients because it is often difficult to obtain, as many patients are hospitalized with psychotic or epileptic symptoms that are uncooperative with all night examination. (3) This study did not analyze the relationship between SD and different subtypes of AE due to small sample. In addition, the current study lacks multimodal imaging; in a single imaging study, it is relatively difficult to directly find the structure and function of the related changes. In the future, large-sample, homogeneous, multimodal, and cohort studies are needed to explore the pathogenesis of SD in AE.

In summary, sleep disturbances occur in more than half of autoimmune encephalitis; a significant practical implication is that testing for AE antibodies should be considered in patients who present sleep disorders and main limbic symptoms. Five risk factors of SD in AE are observed, including smoking, HAMD score, MoCA score, hyperhomocysteinemia, and elevated NSE level. It is essential to timely monitor these factors, which may help to improve diagnosis and prognosis of AE patients. Moreover, a widespread brain networks dysfunction may be the potential neuro-metabolic mechanism of SD in AE. Further larger and prospective studies are needed to clarify and validate the sleep subtypes and pathological mechanisms of SD in AE.

## Data Availability Statement

The original contributions presented in the study are included in the article/**Supplementary Material**. Further inquiries can be directed to the corresponding author.

## Ethics Statement

The studies involving human participants were reviewed and approved by the Ethics Committee of the Beijing Tiantan Hospital that was affiliated to the Capital Medical University of the People’s Republic of China. The patients/participants provided their written informed consent to participate in this study.

## Author Contributions

XL and QW recruited, diagnosed, and assessed patients. XL, TY, XZ, PY, RL, CW, LA, and QW worked on the establishment of the separate databases. XL drafted a significant portion of the manuscript or figures. XZ, RL, CW, LA, and QW reanalyzed and interpreted all final data. All authors contributed to the article and approved the submitted version.

## Funding

The study was financially supported by the National Key R&D Program of China grant (2017YFC1307500), the Capital Health Research and Development of Special grants (2016-1-2011 and 2020-1-2013), the Beijing–Tianjin–Hebei Cooperative Basic Research Program (H2018206435), the Beijing Natural Science Foundation (Z200024, 7192054), the National Natural Science Foundation of China (81771143 and 2018YFC1315201), the Application Research of Capital Clinical Characteristics (No. Z181100001718082), and the Beijing Dongcheng District Outstanding Talent Funding Project (No. 2019DCT-M-18).

## Conflict of Interest

The authors declare that the research was conducted in the absence of any commercial or financial relationships that could be construed as a potential conflict of interest.

## Publisher’s Note

All claims expressed in this article are solely those of the authors and do not necessarily represent those of their affiliated organizations, or those of the publisher, the editors and the reviewers. Any product that may be evaluated in this article, or claim that may be made by its manufacturer, is not guaranteed or endorsed by the publisher.

## Abbreviations

AE, autoimmune encephalitis; SD, sleep disorders; 18F-FDG-PET, 18F-fluorodeoxy-glucose positron emission tomography; NMDAR, N-methyl-D-aspartate receptor; LGI1, leucin-rich glioma inactivated-1; CASPR2, contactin-associated protein-2; GABAB, γ-aminobutyric acid type B; AMPAR, α-amino-3-hydroxy-5-methyl-4-isoxazolepropionic acid receptor; GAD65, glutamic acid decarboxylase 65; PSQI, Pittsburgh sleep quality index; BMI, body mass index; MoCA, Montreal cognitive assessment; HAMD, Hamilton Depression scale; HAMA, Hamilton Anxiety scale; NLR, neutrophil-to-lymphocyte ratio; NSE, neuron-specific enolase; SPM, statistical parametric mapping; FDR, false discovery rate; IQR, interquartile range.
