# Supplementary material for: Risk Factors and Brain Metabolic Mechanism of Sleep Disorders in Autoimmune Encephalitis
Source: Front Immunol. 2021 Nov 24;12:738097. doi: 10.3389/fimmu.2021.738097 (PMC8652207; doi:10.3389/fimmu.2021.738097)
Supplement: Supplementary file 1 [file Table_1.docx]

| **Supplementary Table 1 Comparison of baseline features between patients with PSG and whole cohort** | | | |
| --- | --- | --- | --- |
|  | Patients with PSG (n=28) | Patients in whole cohort (n=121) | *P* value |
| Age, median (IQR), years | 57 (35-66) | 54 (37-64) | 0.526 |
| Male, n (%) | 19 (67.9) | 77 (63.6) | 0.674 |
| Body mass index, mean (standard deviation) | 24.8 (4.8) | 24.5 (3.9) | 0.758 |
| Medical history, n (%) |  |  |  |
| Hypertension | 6 (21.4) | 31 (25.6) | 0.644 |
| Diabetes | 3 (10.7) | 11 (9.1) | 0.728 |
| Smoking | 8 (28.6) | 33 (27.3) | 0.89 |
| Drinking | 5 (17.9) | 28 (23.1) | 0.544 |
| MoCA score, median (IQR) | 19 (14-24) | 20 (15-24) | 0.819 |
| HAMA score, median (IQR) | 8 (4-15) | 10 (4.5-14.5) | 0.623 |
| HAMD score, median (IQR) | 12 (3-17) | 12 (6-17) | 0.546 |
| PSQI score, median (IQR) | 7 (3-11) | 6 (3-9) | 0.491 |
| Tumor, n (%) | 2 (7.1) | 11 (9.1) | 0.742 |
| EEG, n (%) |  |  | 0.734 |
| Slow waves or epileptiform discharges in temporal region | 19 (67.9) | 78 (64.5) |  |
| Others | 9 (32.1) | 43 (35.5) |  |
| Initial MRI results, n (%) |  |  | 0.359 |
| Normal | 14 (50) | 72 (59.5) |  |
| Medial temporal lesions | 14 (50) | 49 (40.5) |  |
| Days from onset to diagnosis | 52 (25-303) | 54 (25-140) | 0.851 |
| Days from diagnosis to PET scans | 6 (2-17) | 6 (2-17) | 0.988 |
| Days from diagnosis to sleep assessment | 10 (6-16) | 9 (6-18) |  |
| First-line Immunotherapy, n (%) |  |  | 0.785 |
| IVMP only | 5 (17.9) | 20 (16.5) |  |
| IVIg only | 6 (21.4) | 21 (17.4) |  |
| IVMP + IVIg | 17 (60.7) | 80 (66.1) |  |

Abbreviations: AE = autoimmune encephalitis; PSG = polysomnography; IQR = interquartile range; MoCA = Montreal cognitive assessment; HAMA = Hamilton anxiety scale; HAMD = Hamilton depression scale; EEG = electroencephalogram; MRI = magnetic resonance imaging; PET = positron emission tomography; IVMP = IV methylprednisolone; IVIG = immunoglobulin.
